# The Virtuous Cycle of a Data Ecosystem

**DOI:** 10.1371/journal.pcbi.1005037

**Published:** 2016-08-04

**Authors:** Bradley Voytek

**Affiliations:** Department of Cognitive Science, Neurosciences Graduate Program, Institute for Neural Computation, and Kavli Institute for Brain and Mind, University of California, San Diego, California, United States of America; National Institutes of Health, UNITED STATES

## Overview

Modern science is creating data at an unprecedented rate, yet most of these data are being discarded. Raw scientific data, when they are published at all, are provided in a very limited form. Large, multidimensional datasets—rich with hidden information—are reduced to summary statistics filtered through limitations imposed by contemporary methods and technologies, and through the biased lens of the originating research group. The massive loss of raw data currently underway, and the lack of a system for discovering them, hinders scientific progress. In this Perspective, I argue that our contemporary limited view of the long-term scientific and medical benefits that could be made possible by data sharing masks the benefits for doing so. This, in turn, makes the costs of data sharing seem higher than they are.

## Introduction

Digital data of all types are being created at an ever-increasing rate, doubling approximately every two years. Annual data creation rates are estimated to reach 44 trillion gigabytes by 2020 [1]. Similarly, the rate at which primary scientific data are being collected is accelerating [2]. This astounding growth in scientific data creation has led to the contemporary discussion of scientific data sharing policies. Many of the criticisms levied against data sharing have focused on practical issues such as the economics and logistics of data storage, technical challenges for doing so, or appropriate attribution of credit [2–9]. In contrast, the arguments in favor of data sharing have focused largely on scientific replication, reproducibility [10], facilitation of collaborative research, and increased citations for publications that share data [11]. This is largely an ethical argument wherein there is an obligation to share data collected using public funds [3–6,12,13].

Rather than focusing on the much-discussed arguments against data sharing—cost, infrastructure, curation, privacy, and attribution/credit concerns—in this Perspective, I outline the overlooked benefits of data sharing: novel remixing and combining as well as bias minimization and meta-analysis. I argue that we must consider the weight of the costs against the true value of the possible benefits. If the decision for any individual researcher, university, or funding agency to implement data sharing policies comes down to a cost—benefit analysis based solely on replication versus storage, the cost—benefit analysis may be artificially tipped in favor of not sharing data caused by overlooking more subtle—but critical—benefits. These hidden benefits of data remixing cannot be appreciated when considering each individual dataset as an independent entity, and thus a richer consideration of those benefits is warranted.

Although there is some evidence that, on the local scale, research groups may not make use of shared data [14], in this Perspective, I outline the ways in which research groups are beginning to take advantage of open data in novel, and sometimes surprising, ways. Rather than arguing for a centralized, large-scale data repository, I am advocating for a more organic development wherein we, institutionally, encourage the growth of a data ecosystem. This can be done via multiple venues, such as the general scientific data sharing sites figshare (https://figshare.com/) or the Dryad Digital Repository (http://datadryad.org/), each of which, in addition to Nature Publishing Group’s recently launched peer-reviewed data sharing journal, *Scientific Data* [15], provides citable Digital Object Identifiers for the data themselves. Such developments are addressing concerns regarding credit and help motivate data curation and contextualization. A data sharing ecosystem provides space for multiple diverse datasets to intermingle to encourage new, multidisciplinary discoveries for current and future scientists.

## Data Sharing Benefits

### Data remixing and combining

One of the potentially most powerful yet underrated benefits of releasing data is the opportunity to reanalyze older data using contemporary methods. There are countless examples of data (broadly construed) being used in novel ways to generate new insights in domains far removed from their original source. Below, I cite four general cases.

#### 1. Reanalyzing old data using new methods

Exoplanets were discovered in decades-old data collected by the Hubble Space Telescope [16]; 19th century naval logbooks were used to extract weather data to model climate change [17]; epigenetic changes in DNA methylation were identified as a function of prenatal exposure to famine as documented by health records preserved from the 1944–45 Dutch Hunger Winter [18]; ink traces of electrophysiological data collected from the human cerebellum in the 1930s and 1940s were digitized and analyzed using modern methods to uncover novel functions of this brain region [19].

#### 2. Text mining for scientific discovery

Text was extracted from millions of books published across hundreds of years to model language evolution and cultural phenomena [20,21]; freeform text from patients writing in online forums was analyzed to aid in clinical discovery [22]; online food recipes were used to uncover cultural taste preferences [23].

#### 3. Data remixing and combination

Data from studies in archeology, criminology, economics, geography, history, political science, and psychology were used to analyze the effect of climate on human conflict [24]; neuroscientific textual information from millions of peer-reviewed papers was compared against human brain gene expression data to identify brain structure, function, and disease relationships [25]; spatial information about the functional relationships of the human brain, as mined from thousands of peer-reviewed papers, was combined with spatial information on human gene expression data to identify novel gene—cognition relationships [26].

#### 4. Semi-automated, or algorithmic, hypothesis generation

Neuronal electrophysiological data were aggregated to study neural diversity [27,28]; research maps of experimental results were created to extract the weight of evidential support or results [29]; possible novel hypotheses were uncovered by analyzing missing connections between scientific topics [25,26,30].

This last point—semi-automated or algorithmic hypothesis generation—has enormous potential to speed scientific discovery. Hypothesis-generation algorithms thrive in an environment rich with independent data sources. The above examples all come from the neurosciences, a field that poses unique challenges for data mining [31]. These projects represent largely independent, parallel efforts operating at different conceptual scales ranging from sub-cellular to psychological. As more neuroscientific datasets become available, it will become increasingly possible to statistically link multiple domains, including gene expression [32], neural diversity [28], functional neuroimaging [33], neural activity [34], and cognition [35]. Once these datasets can be aligned in a common format, hypothesis generation algorithms can be deployed to identify candidate links between genes, neural activity, cognition, and disease.

### Bias minimization and meta-analysis

Another benefit of large-scale data availability is that it could uncover sampling bias by allowing researchers to combine data from multiple studies. For example, sampling bias is rampant in psychology, in which 96% of studies published from the top six psychology journals consisted of data collected from people living in Western, Educated, Industrialized, Rich, and Democratic (WEIRD) societies [36]. Furthermore, many datasets, both human [36,37] and rodent [38], are biased in their gender sampling, calling into question the generalizability of many biomedical findings. By combining data from sources collected from animals of different ages and genders, or people from different cultures, the generalizability of the results can be assessed.

Similarly, unless raw data are shared, access to them is limited to those who collected it (and their collaborators). Given that the vast majority of scientific research is conducted by industrialized societies, this limits the interpretation of those data through a narrower cultural lens. There is ample evidence that culture at all levels affects data collection and interpretation, ranging from the “publish or perish” culture of modern academic science biasing what results are published to larger, more macroscale political and social influences in how findings are contextualized [39–41].

One way of minimizing bias is through meta-analysis. However, these analyses, wherein the results of many peer-reviewed studies are aggregated, are limited by the massive data reduction that results from reporting summary statistics. This data reduction—taking a rich, multivariate dataset and summarizing it for publication using measures of central tendency, confidence intervals, *p*-values, and effect sizes—removes the opportunity for future scientists to apply new algorithms, methods, and transdisciplinary ideas that could yield unforeseen insights and discoveries [42,43]. This is because future reanalyses of existing data are restricted to looking only at whatever summary statistics the authors decided to include in their original manuscripts. Given that the majority of raw scientific data are reported to be inaccessible or lost [44], future opportunities to put historical results in context are limited.

Thus, it is important to ensure that data are discoverable and that access to these data be open—similar to the current PubMed search engine and PubMed Central manuscript repository—to limit the currently large digital cultural divide [37]. Closing this divide allows access to those who may not have sufficient resources to run large-scale experiments on their own. It also opens up the opportunity for broader interpretation and contextualization of those data, as well as democratization of the scientific process through citizen science, which has proved to be a highly successful model such as Foldit [45], EyeWire [46], and Galaxy Zoo [47].

## Conclusion

Modern science is massive in scale; the data we are generating are evidence of our advancing knowledge. The simultaneous growth of data collection techniques [48] along with data aggregation and mining algorithms [49,50] provides an unprecedented opportunity for rapid knowledge discovery [51]. We cannot know what other discoveries lay hidden in our data, similar to how even the most innocuous-seeming scientific results can lead to important breakthroughs. To give but a few examples of this: studying monkey social behaviors and eating habits led to insights into the origins of HIV [52]; research into how algae move toward light paved the way for optogenetics—using light to control neural activity [53]; and black hole research spurred the development of algorithms eventually used as part of the 802.11 specifications ubiquitously used in modern Wi-Fi [54]. The ideas spawned from the above projects (and countless others) could never have been anticipated. They cut across broad research domains well outside their original fields. However, the possibility for a breakthrough can't exist if we base our decision-making on the immediately obvious and predictable outcomes.

Of course, there are concerns for sharing data, and privacy and consent issues surrounding the sharing of human data are complex [55]. Privacy issues are compounded by the fact that even data that have been de-identified can be re-identified [56], so care must be taken to ensure individual privacy until de-identification has been proved to be secure. Nevertheless, encouraging the growth of a data ecosystem should be a priority among scientists. By basing the decision of whether or not to share data solely on whether replication and reproducibility is worth the cost of curation and storage, we are limiting the opportunities for future scientists to make novel use of our data in ways that we could never predict. By sharing the raw data, we can create a virtuous cycle that allows researchers to remix and reanalyze data in new and interesting ways. It is our duty to preserve our data so that future generations will not be hindered by our prejudiced interpretations and analytical limitations.
